# Supplementary material for: Baicalin Relieves LPS-Induced Lung Inflammation via the NF-κB and MAPK Pathways
Source: Molecules. 2023 Feb 16;28(4):1873. doi: 10.3390/molecules28041873 (PMC9966172; doi:10.3390/molecules28041873)
Supplement: Supplementary file 1 [file molecules-28-01873-s001.zip › molecules-2147995-supplementary.pdf]

Supplement Table S1 Effect of baicalin on the number and proportion of leukocyte cell in LPS-induced mice for 6h, 12h and 24h.

Item	Group and time									Unit	Reference range
	Control			LPS			LPS+Baicalin				
	6 h	12 h	24 h	6 h	12 h	24 h	6 h	12 h	24 h		
WBC	6.2	6.1	5.5	12.7	13.1	8.8	7.1	6.0	5.7	10^9/L	0.8-6.8
Lymph	4.1	4.1	3.0	6.4	8.4	5.8	5.2	3.1	3.9	10^9/L	0.7-5.7
Mon	0.3	0.3	0.4	0.7	1.3	0.3	0.5	0.3	0.2	10^9/L	0.0-0.3
Gran	1.8	1.7	2.1	5.6	3.4	2.7	1.4	2.6	1.6	10^9/L	0.1-1.8
Lymph%	66.2	67.5	54.9	50.4	64.1	65.9	73.2	51.7	68.4	%	55.8-90.6
Mon%	4.8	4.5	6.7	5.5	9.9	3.4	7.1	5.7	3.9	%	1.8-6.0
Gran%	29.0	28.0	38.4	44.1	26.0	30.7	19.7	42.6	27.7	%	8.6-38.9
RBC	11.68	12.02	11.40	12.04	11.43	11.76	11.31	11.38	11.89	10^{12}/L	6.36-9.42
HGB	176	183	173	182	172	181	163	174	179	g/L	110-143
HCT	55.5	58.0	54.6	58.2	54.0	57.7	51.6	55.9	57.1	%	34.6-44.6
MCV	47.6	48.3	47.9	48.4	47.3	49.1	45.7	49.2	48.1	fL	48.2-58.3
MCH	15.0	15.2	15.1	15.1	15.0	15.3	14.4	15.2	15.0	pg	15.8-19
MCHC	317	315	316	312	318	313	315	311	313	g/L	302-353
RDW	14.3	14.3	12.9	13.2	13.5	13.6	14.1	14.0	13.2	%	13-17
PLT	1090	1143	1200	1181	1017	1081	976	1039	1154	10^9/L	450-1590
MPV	6.7	7.2	6.7	6.6	6.7	6.6	6.2	6.6	7.0	fL	3.8-6.0
PDW	16.7	16.7	17.0	16.8	16.9	16.4	16.4	16.6	16.8		

[1]CON, control group; LPS, LPS group; LPS+BA, LPS+baicalin group. n=6 in each group; [2]WBC, white blood cell; [3]Lymph, Lyamphocyte; [4]Mon, Monocyte; [5]Gran, neutrophil; [6]RBC, red blood cell; [7]HGB, hemoglobin; [8]HCT, hematocrit; [9]MCV, Mean Corpuscular Volume; [10]MCH, Mean Corpuscular Hemoglobin;[11]MCHC, Mean Corpusular Hemoglobin Concerntration; [12]RDW, red cell distribution width; [13]PLT, Platelets; [14]MPV, Mean Platelet Volume; [15]PDW, platelet distribution width.

$^{a–b}$ Means with different superscript letter differ ($P < 0.05$) within groups.
